# Addition of oh8dG to Cardioplegia Attenuated Myocardial Oxidative Injury through the Inhibition of Sodium Bicarbonate Cotransporter Activity

**DOI:** 10.3390/antiox11091641

**Published:** 2022-08-24

**Authors:** Min Jeong Ji, Kuk Hui Son, Jeong Hee Hong

**Affiliations:** 1 Department of Health Sciences and Technology, Lee Gil Ya Cancer and Diabetes Institute, GAIHST, Gachon University, 155 Getbeolro, Yeonsu-gu, Incheon 21999, Korea; 2 Department of Thoracic and Cardiovascular Surgery, Gachon University Gil Medical Center, Gachon University, Incheon 21565, Korea

**Keywords:** cardioplegia, oxidative stress, 8-hydroxy-2′-deoxyguanosine, sodium bicarbonate cotransporter, reperfusion

## Abstract

The biomarker 8-hydroxy-2′-deoxyguanosine (oh8dG) is derived from oxidized nucleic acids or products of oxidant-mediated DNA damage. Enhanced sodium bicarbonate cotransporter (NBC) activity is caused by reactive oxygen species (ROS) production in ventricular myocytes. Thus, we hypothesized that cardioplegia-solution-mediated ROS generation may be involved in the regulation of NBC activity in cardiomyocytes and that oh8dG treatment may modulate ROS and associated NBC activity. Langendorff-free cardioplegia-arrested cardiac strips and cardiomyocytes were isolated to determine the NBC activity and effects of oh8dG on oxidative-stress-mediated cardiac damage markers. We first determined the histidine-tryptophan-ketoglutarate (HTK) solution mediated NBC activity in cardiac strips and cells. The oh8dG treatment attenuated NBC activity in the electroneutral or electrogenic form of NBC. Additionally, exposure to HTK solution induced ROS, whereas co-administration of oh8dG attenuated ROS-mediated NBC activity, reduced ROS levels, and decreased the expression of apoptotic markers and fibrosis-associated proteins in cardiac cells. The oh8dG-administrated cardiac tissues were also protected from enhanced HTK-induced damage markers, heat shock protein 60 and polyADP-ribose. Our results show that oh8dG has a protective role against myocardial oxidative damage and provides a useful treatment strategy for restoring cardiac function.

## 1. Introduction

To decrease primary graft failure and postoperative myocardial dysfunction, donor heart preservation is an important process during heart transplantation [1,2]. Extracted donor hearts are exposed to ischemia, which leads to the deletion of the adenosine triphosphate (ATP) stores that initiate anaerobic metabolism and intracellular acidosis [3,4]. Increased intracellular enhanced activity of the sodium–hydrogen exchanger (NHE) leads to sodium influx into myocardial cells [3,4]. After the transplantation procedure, reperfusion of the donor heart rapidly initiates the washout of hydrogen (H^+^) to the interstitial space. It creates a huge gradient of H^+^, which leads to further sodium influx through NHE [5]. The influx of sodium leads to upregulation of the sodium–calcium exchanger (NCX) in reverse mode, which eventually results in the import of calcium into myocardial cells [5]. Intracellular calcium overload enhances myocardial cell death by increased reactive oxygen species (ROS) levels, hyper-contracture, and apoptosis [3,4,5]. To decrease ischemia–reperfusion injury, retrieved organs are generally preserved in cold (4 °C) organ-preserving solutions [6]. One of the most frequently used organ-preserving solutions is the histidine-tryptophan-ketoglutarate (HTK) solution [7]. As antioxidants, histidine, and tryptophan play roles in decreasing ROS generation [8,9]. However, it is known that HTK solution during hypothermic organ preservation is not effective in preventing ROS generation [10,11]. Several animal studies have shown that NHE inhibitors or NCX inhibitors decrease reperfusion injury in cardiomyocytes by delaying pH normalization and decreasing calcium accumulation in the cytosol [12,13]; however, these inhibitors are not available for clinical use [14]. Sodium-bicarbonate cotransporters (NBCs), like NHE, are acid-extruders involved in controlling intracellular pH in myocardial cells [15]. It is also known that activation of NBCs leads to calcium overload during reperfusion [16].

Moreover, angiotensin II (AngII)-mediated alkalization is mediated by NBC activity in rat ventricular myocytes [17]. AngII enhances NADPH oxidase-mediated ROS production and, subsequently, activates electroneutral NBC activity but not electrogenic NBC in cardiac cells [18,19]. Thus, we hypothesized that cardioplegia solution-mediated ROS generation is involved in regulating NBC activity in cardiomyocytes.

The biomarker 8-hydroxy-2′-deoxyguanosine (oh8dG) is a naturally driven oxidized nucleic acid or a product of oxidant-mediated DNA damage [20]. Various studies have reported that oh8dG also exhibits anti-oxidative effects and is a biomarker of oxidative stress in numerous tissues, and administration of exogenous oh8dG involves the inhibition of inflammatory cascade including Rac-GTP, ROS, and NF-κB signaling [21,22,23,24,25,26,27,28,29,30,31,32,33].

ROS-mediated mechanisms are suppressed by the administration of oh8dG in various diseases such as gastritis [34], obesity [35], or ultraviolet B-induced skin damage [24]. Accumulating evidence of an exogenous oh8dG anti-oxidative or anti-inflammatory effect drives to determine the role of oh8dG against oxidants in cardioplegia-solution-exposed cardiac tissues. In addition, exogenous oh8dG is a non-mutagenic compound and non-corporates into DNA [21,32,36,37]. In this study, we determined cardiac NBC activity by administering cardioplegia solution and verified the role of exogenous oh8dG on the regulation of NBC activity in cardiac tissues. Thus, ROS-mediated NBC activity was determined and the cardiac-tissue-protective role of cardioplegia solution with exogenous oh8dG was evaluated. oh8dG, a potential target drug, is considered a protective supplement against cardioplegia solution-mediated cardiac oxidative damage and is a useful treatment strategy for restoring cardiac function.

## 2. Materials and Methods

### 2.1. Animals

All animal care and experimental procedures for heart isolations from mice followed Gachon University guidelines and were approved by the Gachon Animal Care and Use Committee of Gachon University (GACUC, LCDI-2016-0037). Wild-type mice (C57BL6/N, male, eight weeks old, Orient Bio, Sungnam, Korea) were housed in the pathogen-free animal facility at the core of the Lee Gil Ya Cancer and Diabetes Institute, Gachon University (Incheon, Korea). Wild-type mice were housed individually in ventilated mouse cages under controlled humidity (50%) and temperature (21.4 °C).

### 2.2. Cardioplegia Infusion and Cardiac Strip Preparation

All procedures were performed as previously described [38] and using the Langendorff-free cardioplegia method [39]. Mice at 8-weeks old were sedated with 5% isoflurane gas, and the chest was opened to expose the entire thorax. The right atrial auricle was excised, and cardioplegia solution (histidine-tryptophan-ketoglutarate solution, HTK) was pumped into the left ventricle using a 23G needle at 1 mL/min per gram of body weight. Wild-type mice were euthanized using isoflurane (Hana Pharm. Co., Ltd., Seoul, Korea). The heart of each mouse was harvested, and the left ventricle (LV) was separated. The isolated LV was minced into small pieces (~100 × 150 μm) on ice. The minced samples were transferred to an EP-Tube and stored in physiological salt solution (PSS) containing [mM] 140 NaCl, 10 HEPES, 10 D-glucose, 5 KCl, 1 MgCl_2_, and 1 CaCl_2_ (adjusted pH 7.4, 300 mOsm) until use. Isolated cardiac strips were incubated in HTK cardioplegia solution (histidine-tryptophan-ketoglutarate, Chemie GMBH, Germany) with or without 8-hydroxy-2′-deoxyguanosine (oh8dG, provided by Myung-Hee Chung [40], and obtained from Sigma Aldrich (H5653, St. Louis, MO, USA)) until needed for subsequent experiments on ice.

### 2.3. Isolation of Single Cardiomyocytes

Cardiomyocytes isolation was performed according to a previously described experimental protocol [38,39]. The LV (8-week-old mice) was injected with 10 mL EDTA buffer. The EDTA buffer was composed of [mM] 130 NaCl, 5 KCl, 10 HEPES, 10 Taurine, 0.5 Na_2_PO_4_, 10 D-glucose, 10 (2,3)-butanedione monoxime (B0753, BDM, Sigma Aldrich, St. Louis, MO, USA), and 5 EDTA. Then, 3 mL perfusion buffer was injected. The perfusion buffer was composed of [mM] 130 NaCl, 5 KCl, 10 HEPES, 10 Taurine, 0.5 Na_2_PO_4_, 10 D-glucose, 10 BDM, and 1 MgCl_2_. The 30 mL collagenase buffer containing 0.5 mg/mL collagenase type II (17101515, Thermo Scientific, Waltham, MA, USA) and 0.05 mg/mL protease XIV (P5147, Sigma Aldrich) was injected into the LV until the heart became soft and reached a transparent status. After mincing the cardiac strips to 1-mm^3^, they were transferred into a new conical tube with a stop buffer containing 5% FBS and filtered using a 100 μm strainer. The sample was centrifuged at 300× *g* for 15 min at 4 °C, and cardiomyocytes were obtained from the supernatant and stored on ice until use.

### 2.4. ROS Staining

Isolated cardiomyocytes, cardiac strips, and cardiac tissues were attached to poly-L-lysine (P8920, Sigma-Aldrich)-coated coverslips for 15 min at room temperature. Next, 10 μM 2′, 7′-Dichlorofluorescin diacetate (DCFDA) was loaded for 15 min and then rinsed with 1× DPBS. Confocal images were obtained at 488 nm using a Zeiss LSM 700 confocal microscope (Fluo-view, Carl Zeiss, Oberkochen, Germany). Images were collected from five separate preparations of cardiac tissue, and the results are presented as the average from all experiments.

### 2.5. Cell Culture and DNA Transfection

Human HEK293T (H.T), lung fibroblast MRC5, lung adenocarcinoma A549 cells were purchased from ATCC (Washington, DC, USA). Cells were cultured in Dulbecco’s Modified Eagle Medium containing 10% fetal bovine serum (FBS) and 100 U/mL penicillin-streptomycin antibiotics at 37 °C in 5% CO_2_/95% air. Human NBCe1-B (an electrogenic form of sodium bicarbonate cotransporter, encoded by *SLC4A4*) and NBCn1 (electroneutral form of sodium bicarbonate cotransporter, encoded by *SLC4A7*) clones were provided by Shmuel Muallem (National Institutes of Health, Bethesda, MD, USA). Plasmid DNAs (1 μg/μL) were mixed with 200 μL Jet Prime Buffer (B200225, Polyplus-transfection, Illkirch-Graffenstaden, France). Transfection reagent (4 mL) (21Y0910L1, Polyplus-transfection) was incubated with mixed Jet prime buffer and DNA for 10 min in the dark. Incubated DNAs were transferred into H.T-cultured plates, and all procedures were performed in accordance with the protocol of the manufacturer (Polyplus-transfection). MRC5 cells were used for determination of cell viability, and A549 cells were used for determination of native NBC activity. S0859 (18497, Cayman, Ann Arbor, MI, USA) was used for the specific inhibition of NBC activity.

### 2.6. Measurement of Na^+^-HCO_3_^−^ Cotransporter (NBC) Activity

The fluorophore 2′-7′-Bis-(carboxyethyl)-5-(and-6)-carboxyfluorescein (0061, BCECF-AM, Teflabs, Austin, TX, USA) was used to measure intracellular pH changes in H.T cells and isolated cardiomyocytes at dual excitation wavelengths (440 and 495 nm) and an emission wavelength (530 nm). Then, 5-(N-Ethyl-N-isopropyl) amiloride (1154-25-2, EIPA, Sigma), a selective inhibitor of NHE, was mixed with HCO_3_^-^ solution to measure only NBC activity. H.T cells and cardiomyocytes were loaded onto coverslips with a mixture of 0.05% Pluronic F-127 and 6 μM BCECF-AM perfused with PSS. NBC activity was measured by incubating the cells with CO_2_-saturated HCO_3_^−^-containing media with EIPA, followed by acidification with Na^+^-free HCO_3_^−^- buffered media. The emitted fluorescence was monitored using a CCD camera (Photometrics) and analyzed using a MetaFluor system (Molecular Devices). All BCECF fluorescence images were gained at 1 sec intervals.

### 2.7. Western Blotting

Cardiac strips isolated from mice were stimulated with HTK cardioplegia solution and oh8dG. Proteins were isolated from cardiac strips using lysis buffer containing (mM) 20 Tris, 150 NaCl, 1% Triton X-100, 2 EDTA, and a protease inhibitor cocktail; a Bradford assay (Quick Start Bovine Serum Albumin standard, 5000207, Bio-Rad, Hercules, CA, USA) was used to quantify the concentration. Protein samples were denatured in sodium dodecyl sulfate (SDS) sample buffer at 37 °C for 30 min. Denatured protein samples (30 μg) were subjected to SDS-polyacrylamide gel electrophoresis. Proteins were transferred to PVDF membrane and incubated primary antibodies (1:1000 dilution), NBC1e1 (ab187511, Abcam, Cambridge, UK), NBCn1 (ab82335, Abcam), connective tissue growth factor (ac365970, CTGF, Abcam), and β-actin (A3854, Sigma, St. Louis, MO, USA) and then visualized with horseradish-conjugated secondary antibodies (1:2000 dilution for mouse and rabbit IgG) using an enhanced chemiluminescence solution (Thermo Fisher Scientific, Waltham, MA, USA).

### 2.8. Reverse Transcription-Polymerase Chain Reaction (RT-PCR)

Total RNA was extracted from the mouse cardiac tissues using the Hybrid-Ribo^Ex^ extraction system (301-001, GeneAll, Seoul, Korea) in accordance with the instructions of the manufacturer. Isolated RNA was quantitated using the Spectrophotometer ND-1000 (Thermo Fisher Scientific), and cDNA was amplified using the TOPscript RT-PCR kit from Enzynomics (RT410S, Daejeon, Korea). The primers and PCR cycling protocol were as follows: mouse Bcl-2 (forward) GCA TCT TCT CCT TCC AGC CTG and (reverse) GAC GCT CTC CAC ACA CAT GAC; mouse p53 (forward) CAT GGC CAT CTA CAA GAA GTC and (reverse) GAA CAT CTC GAA GCG TTT ACG; denaturation at 95 °C (5 min), followed by 35 cycles of 95 °C (1 min), an annealing step (1 min), and an extension step at 72 °C (1 min). The final extension step was performed at 72 °C (10 min).

### 2.9. Transferase (TdT)-Mediated dUTP Nick End Labeling (TUNEL) Assay

Frozen cardiac tissues of mice were sectioned on a glass slide at a thickness of 10 μm. The TUNEL assay was performed in accordance with the protocol of the manufacturer, using a Transdetect In Situ Fluorescein TUNEL Cell Apoptosis Detection kit (FA201-02, TransGen Biotech Co., LTD, China). The slides were three times washed with 1× Dulbecco’s phosphate-buffered saline (LB 001-02, DPBS, Kyungsan, Korea) for 10 min and fixed with 4% paraformaldehyde for 30 min at room temperature. The 1× labeling solution (FA201-02, TransGen Biotech Co., LTD, Beijing, China) was added to the surface of the glass slide with tissue attached and incubated at 37 °C in the dark for 1 h. The glass slides were rinsed with 1× DPBS and DAPI-included fluoromount-G (17984-24, Electron Microscopy Sciences, Hatfield, PA, USA) for 5 min. Confocal images were obtained at 358 nm using a Zeiss LSM 700 confocal microscope (Fluo-view, Carl Zeiss). Images were collected from five separate preparations of the cardiac tissues.

### 2.10. Immunostaining

Frozen cardiac tissues of mice were sectioned on the slide glass at a thickness of 10 μm. Slides were fixed with 4% paraformaldehyde or chilled methanol for 10 min. Heat shock protein 60 (ab46798, HSP60, Abcam) and poly (ADP-Ribose) (SM1398, PAR, ORIGENE, Rockville, MD, USA) antibodies were diluted at 1:100 and used to treat fixed samples for 24 h. Secondary antibodies tagged with FITC (for PAR, 1:200 dilution) and rhodamine (for HSP60, 1:200 dilution) were attached, and confocal images were obtained. Images were obtained using an LSM 700 Zeiss confocal microscope (Fluo-view, Carl Zeiss) at 488 nm (FITC), 530 nm (rhodamine), and 358 nm (DAPI for nucleus staining) and analyzed with ZEN software (ZEN 2009 light edition, Carl Zeiss). Images were collected from five separate preparations of cardiac tissue, and the results were averaged from all experiments.

### 2.11. MTT Assay

MRC5 cells were cultured in 96-well plates for 24 h and various dose of oh8dG was added for 1 h. Tetrazolium bromide (MTT; 298-93-1, Merck, Burlington, MA, USA) dye was mixed with 1 mL of PBS, and the cells were treated with 100 μL of MTT dye and incubated for 2 h in the dark. The medium was carefully aspirated from the plate and added 100 µL of DMSO, and the absorbance of sample was measured at 570 nm using a fluorescence microplate reader (VICTOR X3, PerkinElmer, Waltham, MA, USA).

### 2.12. Statistical Analysis

Experimental results are expressed as the mean ± standard error of the mean (SEM), and standard deviation (SD) was also represented. Experiment was performed more than three times and represented in figure legends. Differences between the mean values of the two samples were analyzed using the Student’s t-test. Significance was determined by analysis of variance in each experiment (* *p* < 0.05, ** *p* < 0.01, and *** *p* < 0.001).

## 3. Results

### 3.1. Treatment of oh8dG Attenuated HTK-Mediated ROS Level in Cardiac Strips and Isolated Cardiomyocytes

**Figure 1:**
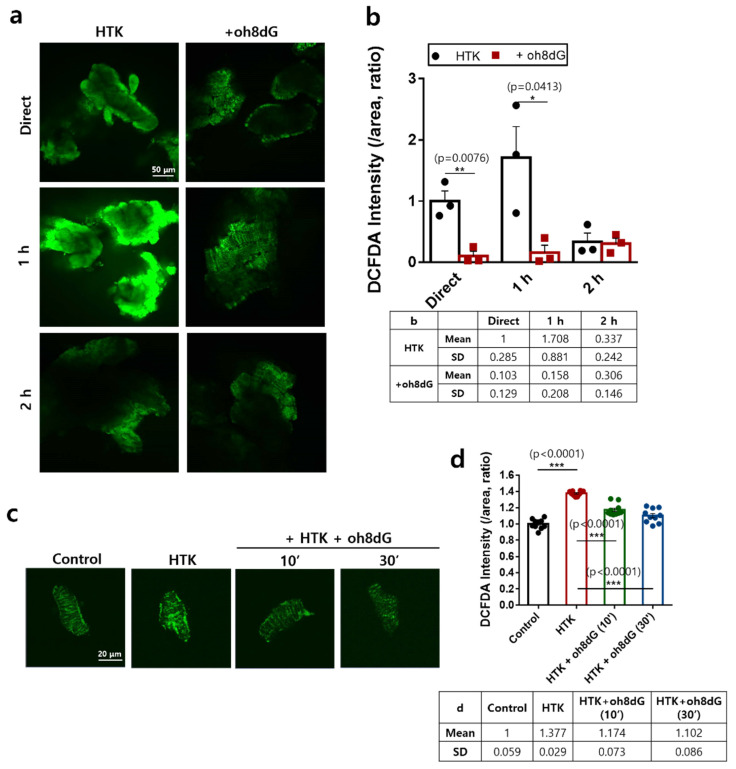
Treatment of oh8dG attenuated HTK-mediated ROS level in cardiac strips and isolated cardiomyocytes. (**a**) DCFDA staining in cardiac strips after cardioplegia and 100 μg/mL oh8dG treatment for 1 and 2 h. Direct cardioplegia arrest with HTK solution using Langendorff-free cardioplegia method for 1 min. The scale bar represents 50 μm. (**b**) Analysis of normalized intensity (total intensity/measuring area) of DCFDA. Bars present mean ± SEM (*n* = 4~6 * *p* < 0.05, ** *p* < 0.01, and *** *p* < 0.001). The table represents mean and SD value. (**c**) DCFDA staining in isolated cardiomyocytes after cardiac arrest with cardioplegia solution (HTK) using Langendorff-free cardioplegia method for 1 min and 100 μg/mL oh8dG was treated for 10 min and 30 min on ice. The scale bar represents 20 μm. (**d**) Analysis of normalized intensity (total intensity/measuring area) of DCFDA. Bars present mean ± SEM (*n* = 10, *** *p* < 0.001). The table represents mean and SD value.

Cardioplegia-induced oxidative injury results in cardiomyocytes apoptosis [41,42]. To determine the anti-oxidative role of exogenous oh8dG, we measured ROS levels using DCFDA fluorescence in cardioplegia solution (HTK)-exposed cardiac strips. DCFDA fluorescence was enhanced in HTK-exposed cardiac strips for 1 h and attenuated by the administration of exogenous oh8dG (Figure 1a,b). The HTK-exposed cardiac strips for 2 h were exhausted of DCFDA fluorescence with or without oh8dG (Figure 1a,b). We also confirmed the anti-oxidative role of oh8dG in cardiomyocytes isolated from the mice. Isolated cardiomyocytes were easily damaged by HTK treatment. Thus, the exposure time of HTK was maintained within 30 min, similar to the experimental condition in cardiac strips, which is available in within 2 h. The improved DCFDA fluorescence in HTK-treated cardiomyocytes was attenuated by treatment with exogenous oh8dG (Figure 1c,d). To verify the toxicity of exogenous oh8dG, we performed an MTT assay (Supplementary Figure S1). There was no effect of oh8dG on cell viability. These results indicate that exogenous oh8dG attenuated HTK-mediated ROS levels in cardiac tissues and cells.

### 3.2. The Treatment of oh8dG Attenuated ROS-Mediated NBC Activities

**Figure 2:**
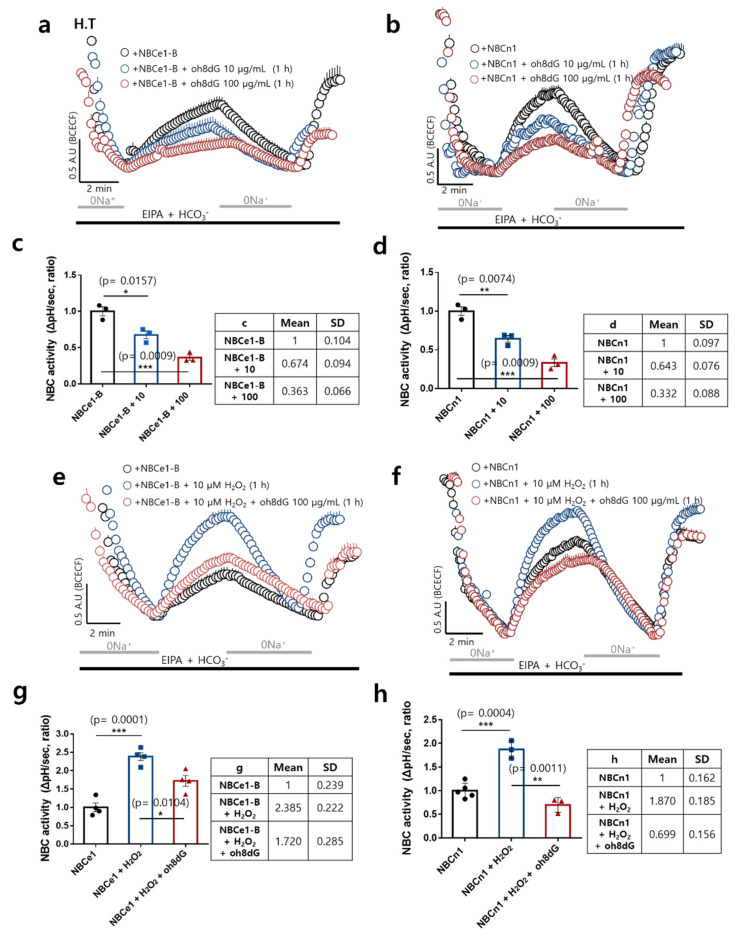
Treatment of oh8dG attenuated ROS-mediated NBC activities. NBC activity was assessed by measuring changes in pH_i_ transfected with NBCe1-B (**a**) and NBCn1 (**b**) plasmids in the presence or absence of oh8dG (10 and 100 μg/mL) for 1 h in HEK293T (H.T) cells. (**c**,**d**) The bars represent mean ± SEM (*n* = 3, * *p* < 0.05, ** *p* < 0.01, and *** *p* < 0.001). The table represents mean and SD value. NBC activity was assessed by measuring 10 μM H_2_O_2_–mediated changes in pH_i_ in HEK293T cells, which were transfected with NBCe1-B (**e**) and NBCn1 (**f**) plasmids in the presence or absence of pre-incubation with 10 μM H_2_O_2_ and oh8dG (100 μg/mL) for 1 h. (**g**,**h**) The bars present mean ± SEM (*n* = 3~5, * *p* < 0.05, ** *p* < 0.01, and *** *p* < 0.001). The table represents mean and SD value.

To verify the role of NBC activity in response to ROS and the anti-oxidative effect of oh8dG, cells were transfected with two types of NBC, NBCe1-B, and NBCn1. NBC-transfected H293T cells were treated with oh8dG for 1 h, and the effective dose of oh8dG was determined. Both 10 and 100 μg/mL of oh8dG attenuated the NBC activities independently of the type of NBC, electrogenic and electroneutral (Figure 2a–d). Then, 100 μg/mL of oh8dG was suggested [28], and we also determined the working dose of oh8dG (100 μg/mL) to evaluate the role of oh8dG. We determined the ROS-modulated NBC activity. NBC-overexpressing cells were stimulated with H_2_O_2_ with or without oh8dG. Treatment with H_2_O_2_ induced two~three-fold high NBC activity in both NBCs, and the co-administration of oh8dG dramatically attenuated NBC activity (Figure 2e–h). We also confirmed the modulatory role of H_2_O_2_ on NBC activity in native NBC-expressed A549 cells. NBC activity was enhanced by the treatment of H_2_O_2_, whereas H_2_O_2_-mediated NBC activity was attenuated by the presence of specific NBC inhibitor S0859 (Supplementary Figure S2). NBC activity was dose-dependently inhibited by the oh8dG treatment (Supplementary Figure S3). These results suggested that NBC activity was directly enhanced by treatment with ROS, and these enhanced activities were attenuated by exogenous oh8dG treatment.

### 3.3. HTK-Exposed Cardiac Tissues Enhanced NBC Activity, Attenuated by the Treatment of oh8dG

**Figure 3:**
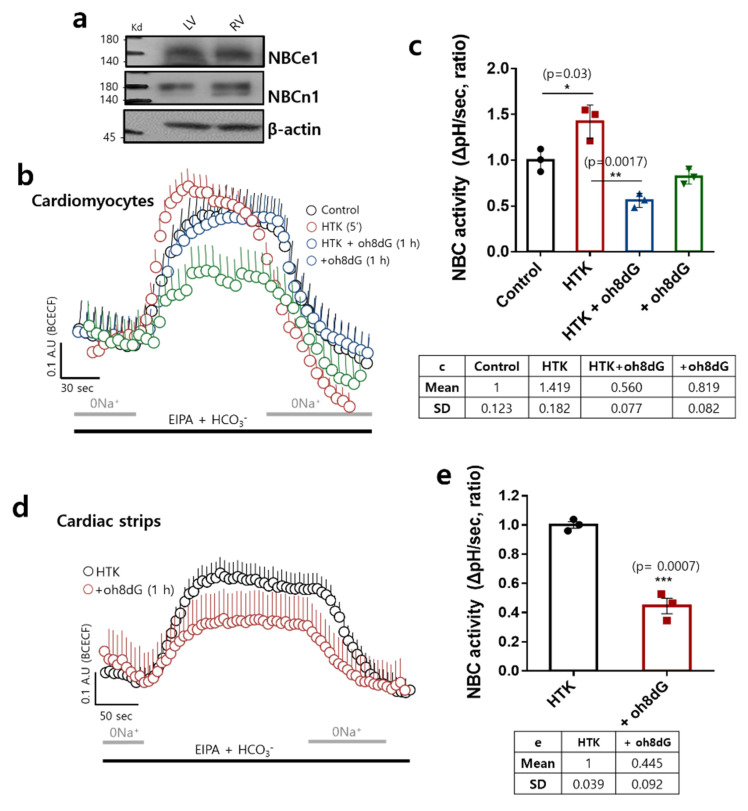
HTK-exposed cardiac tissues enhanced NBC activity, attenuated by the treatment of oh8dG. (**a**) Protein expression of NBCe1 and NBCn1 in left and right ventricle (LV and RV, respectively) cardiac strips of mice (*n* = 6). β-actin was used as the loading control. (**b**) NBC activity was assessed by measuring changes of pH_i_ with or without pre-incubation of 100 μg/mL oh8dG for 1 h in cardiomyocytes on ice. HTK solution was treated for 5 min. (**c**) The bars present mean ± SEM (*n* = 3, * *p* < 0.05, and ** *p* < 0.01) in cardiomyocytes. The table represents mean and SD value. (**d**) NBC activity in cardiac strips during cardioplegia (HTK)-induced arrest for 1 min. After cardiac arrest, 100 μg/mL oh8dG was pre-incubated for 1 h. (**e**) The bars present mean ± SEM (*n* = 3, *** *p* < 0.001) in cardiac strips. The table represents mean and SD value.

To verify the modulatory role of HTK-induced ROS in NBC activity in cardiac tissues, two types of NBC expression were determined. The left and right ventricles of the cardiac tissues were expressed in both NBCs (Figure 3a). Exposure to the HTK solution stimulated NBC activity in isolated cardiomyocytes (Figure 3b,c). Co-stimulation of HTK with oh8dG attenuated HTK-mediated NBC activity (Figure 3b,c). We then confirmed NBC activity in HTK-exposed isolated cardiac strips. oh8dG also attenuated NBC activity (Figure 3d,e). These results indicate that HTK exposure enhanced ROS production and enhanced NBC activity, which was attenuated by exogenous oh8dG administration.

### 3.4. Treatment of oh8dG Attenuated DNA Fragmentation and Expression of the Apoptotic Marker in HTK-Exposed Cardiac Tissues

**Figure 4:**
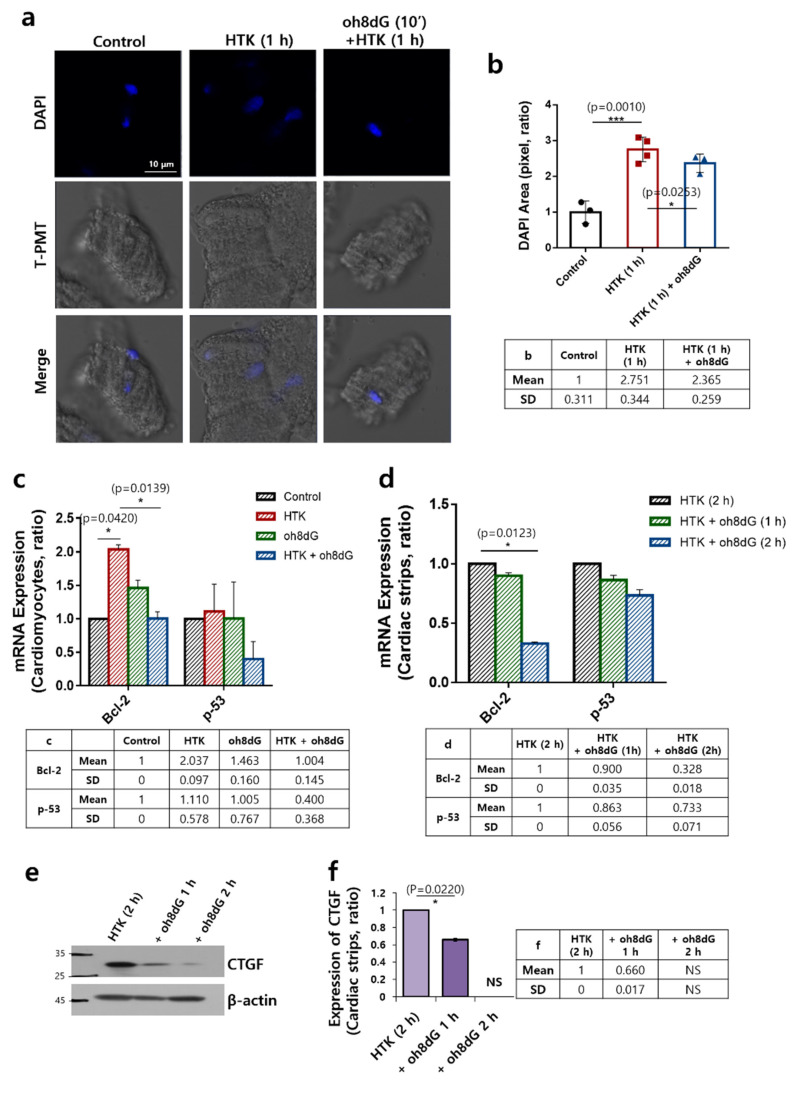
Treatment of oh8dG attenuated DNA fragmentation and expression of the apoptotic marker in HTK-exposed cardiac tissues. (**a**) DAPI (nucleus, blue) staining and T-PMT (gray) of isolated cardiomyocytes in pre-incubation of HTK solution for 1 h and 100 μg/mL oh8dG for 10 min. The scale bar represents 10 μm. (**b**) Analysis of total intensity of DAPI. Bars present mean ± SEM (*n* = 3, * *p* < 0.05 and *** *p* < 0.001). The table represents mean and SD value. (**c**) mRNA expression of Bcl-2 and p-53 in HTK-treated cardiomyocytes for 5 min in the absence and presence of pre-incubation of 100 μg/mL oh8dG for 1 h on ice. The bars present mean ± SEM (*n* = 3, * *p* < 0.05). The table represents mean and SD value. (**d**) mRNA expression of Bcl-2 and p-53 in HTK-treated cardiac strips for 2 h in the absence and presence of 100 μg/mL oh8dG for 2 h on ice. The bars present mean ± SEM (*n* = 3, * *p* < 0.05). NS; not significant. The table represents mean and SD value. (**e**) Protein expression of CTGF in cardiac strips of mice treated with cardioplegia solution (HTK) and 100 μg/mL oh8dG for 1 h and 2 h on ice. β-actin was used as the loading control. (**f**) Analysis of band intensity of CTGF in cardiac strips. The bars present mean ± SEM (*n* = 3, * *p* < 0.05). The table represents mean and SD value.

Ischemia–reperfusion injury-mediated cellular apoptosis is mediated by caspase-3 activation [43] and, subsequently, induces DNA fragmentation [44]. Figure 4a shows an enhanced DAPI staining area that was observed in the HTK-treated cardiomyocytes. HTK-mediated DNA fragmentation was reduced by exogenous oh8dG treatment (Figure 4a,b). The HTK-induced increase in the apoptotic marker Bcl-2, but not p-53, was reduced by oh8dG treatment (Figure 4c,d). The cardiac injury induces the fibrosis factor connective tissue growth factor (CTGF) [45,46]. The oh8dG treatment reduced the expression of CTGF (Figure 4e,f). Isolated cardiac strips were exposed to HTK during cardiac arrest. Thus, the inhibitory effect of oh8dG on CTGF expression was assessed using HTK-exposed cardiac strips as a control. These results indicated that HTK-mediated DNA fragmentation and expression of the apoptotic marker Bcl-2 were attenuated by exogenous oh8dG treatment, with a decrease in CTGF.

### 3.5. Treatment of oh8dG Attenuated HTK-Mediated Cardiac Destructive Signals

**Figure 5:**
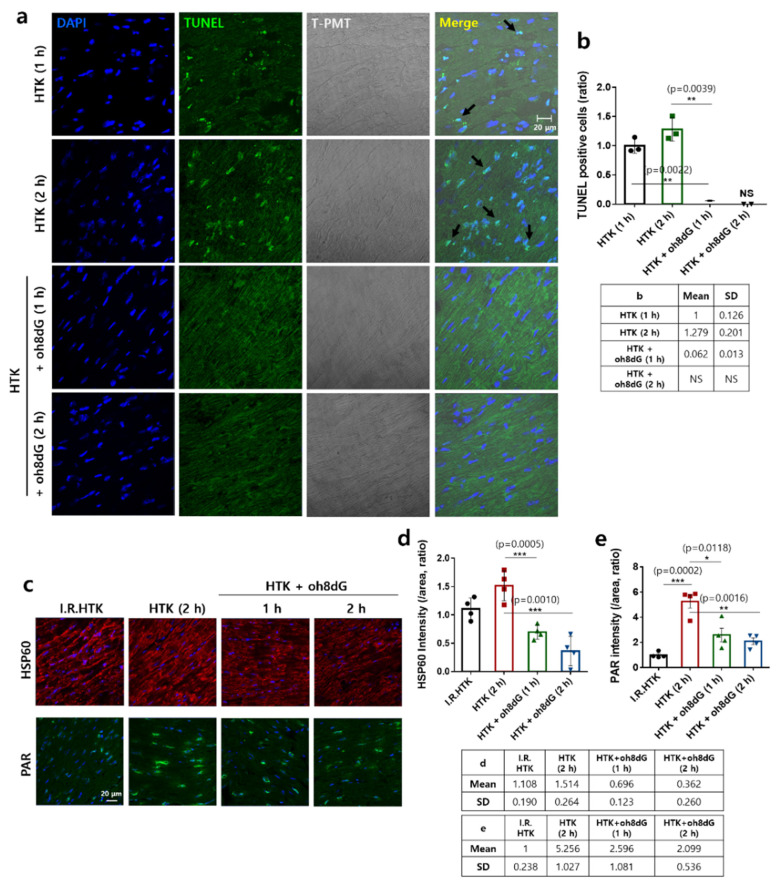
Treatment of oh8dG attenuated HTK-mediated cardiac destructive signals. (**a**) DNA fragmentation was measured by a TUNEL assay on the heart section of mice. The heart was treated with HTK solution for 1 h and 2 h, respectively, including 100 μg/mL oh8dG at different times. Blue (DAPI, nucleus), green (TUNEL), and gray (T-PMT, tissue image). The scale bar represents 20 μm. (**b**) Analysis of TUNEL positive cells (%) normalized by assessing total intensity/measuring area of FITC (green). Bars present mean ± SEM (*n* = 3, * *p* < 0.05). NS; not significant. The table represents mean and SD value. (**c**) Immunostaining of HSP60 (red), PAR (green), and nucleus (DAPI, blue) in cardiac strips of mice. I.R.HTK; immediately removed HTK (cardioplegia-induced arrest, 1 min). The heart was treated with HTK solution for 2 h, and 100 μg/mL oh8dG was treated for 1 h and 2 h, respectively on ice. The scale bars represent 20 μm. Analysis of normalized intensity (total intensity/measuring area) of HSP60 (**d**) and PAR (**e**). Bars present mean ± SEM (*n* = 4, * *p* < 0.05, ** *p* < 0.01, and *** *p* < 0.001). The table represents mean and SD value.

The exogenous oh8dG showed anti-oxidative and anti-apoptotic effects in cardiac tissues and cells. To verify the tissue-protective role of exogenous oh8dG in cardiac apoptotic death, we performed a TUNEL assay. The number of TUNEL-positive cardiac tissues was increased by HTK exposure in a time-dependent manner. The co-administration of oh8dG dramatically reduced the number of TUNEL-positive cells (Figure 5a,b). Cellular stress and apoptotic signals induce plasma membrane localization of the mitochondrial protein HSP60 [47]. In addition, PAR production is activated by DNA damage and stress [48]. HTK-exposed cardiac tissue showed enhanced membrane expression of HSP60 (Figure 5c). We verified the role of exogenous oh8dG in stress/injury-associated protein expression. Co-administration of oh8dG dramatically attenuated HTK-mediated HSP60/PAR expression (Figure 5c–e). These data indicate that the co-administration of HTK and exogenous oh8dG has a protective effect against HTK-associated cardiac destructive signals.

## 4. Discussion

In the present study, we addressed that exposure to HTK solution in cardiac cells induced ROS and co-administration of exogenous oh8dG attenuated ROS-mediated NBC activity and reduced expression of apoptotic markers and fibrosis-associated protein CTGF. The exogenous oh8dG-administrated cardiac tissues were protected from the HTK-induced damage markers HSP60 and PAR. Our results addressed exogenous oh8dG as a protective supplement against potential HTK-solution-mediated oxidative damage. Thus, our observations in cardiac tissues are consistent with previous studies in various experimental systems showing the protective role of exogenous oh8dG against oxidative or inflammatory signals [21,22,23,24,25,26,27,28].

NBC activity is modulated by various cellular stimulants, such as ROS [18], acidic [49], hypoxia [50], and inflammatory cytokines [49]. Our previous study showed that HTK-induced arrest mediated more acidic intracellular circumstances through the involvement of sodium-potassium-chloride cotransporter Nkcc1 and chloride-bicarbonate exchanger Slc26a6 in the *db/db* diabetic mouse model’s heart than in wild-type mice during reperfusion [38].

**Figure 6:**
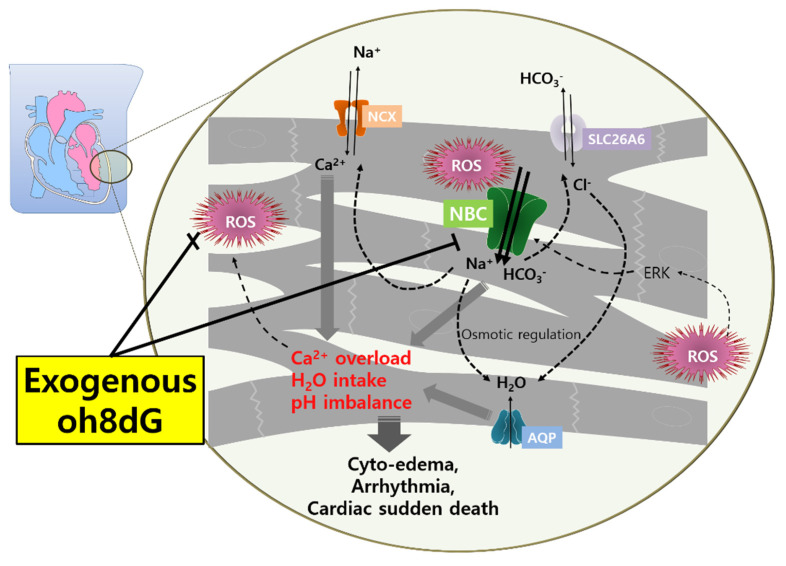
Schematic model of enhanced NBC and its coordinated channels and transporters in HTK-exposed cardiomyocytes and preserving effect of exogenous oh8dG. HTK-solution-exposed cardiac tissues produce oxidative signals. Oxidants enhance cardiac NBC activity through the involvement of ERK [51] and, subsequently, induce convergent involvement of NCX, CBE (SLC26A6), and aquaporin (AQP), which cause calcium overload and cyto-edema during reperfusion [16]. Cardiac calcium overload also mediates ROS generation [52]. Thus, the modulation of NBC activity may be a potential strategy against cardiac damage such as arrhythmic events or cardiac sudden death. The application of exogenous oh8dG may attenuate HTK-mediated NBC hyperactivity and the associated damage markers.

Although NBC is considered an intracellular pH regulator, it has not been revealed whether HTK is involved in the activity of NBCs. The present study showed that NBC activity was increased by HTK-mediated ROS generation. Enhanced NBC activity may mediate sodium and bicarbonate uptake and, subsequently, coordinate ion transport, such as calcium and chloride. We speculate that the accumulation of sodium and bicarbonate through enhanced NBC may mediate the activation of the chloride/bicarbonate exchanger (chloride influx and bicarbonate efflux) and NCX exchanger (calcium influx and sodium efflux). In addition, enhanced sodium and chloride may facilitate water influx to maintain cellular osmotic power (Figure 6). Therefore, calcium overload, enhanced sodium/chloride level, and subsequent water intake occur in the cytosol of cardiomyocytes and, subsequently, induced cytotoxic edema and impaired cardiomyocytes’ function. Similarly, it has been addressed that the angiotensin II-mediated modulation of NBC and pathological output of associated ion transporters in cardiopathies [51]. Although experimental limitations were revealed, our results provide new potential targets to maintain cardiac function after cardiac surgery or heart transplantation using the HTK solution. Modulation of NBC is essential, to protect the heart during the reperfusion period of cardiac surgery or transplantation, by decreasing ROS generation.

ROS-mediated NBC activities, including NBCn and NBCe, were attenuated by treatment with exogenous oh8dG. The signaling of oh8dG has been known in the inactivation of Ras in cancer cells [53]. Ras signaling is involved in migration in various cellular systems, such as vascular smooth muscle cells, endothelial cells, and several cancers [54,55,56,57]. In addition, NBCn is known to provide a migratory module in various cell types, such as vascular smooth muscle cells, breast cancers, and synoviocytes [49,58,59]. Currently, there is no direct evidence for an association between Ras and NBCn. In this regard, the protein–protein network between NBCn and Ras signaling is an attractive and changeable issue for future studies.

The protective role of exogenous oh8dG in various inflammatory and oxidative stress-mediated diseases has been highlighted in different studies [21,23,29,30,31,32,33]. Our results indicate that long-term exposure to HTK solution mediated the enhancement of tissue damage markers. Cardiac tissues should be maintained and protected from damage signals during cardioplegia exposure. The static hypothermic preservation method is a low-cost and standard method for heart transplantation [60]. HTK has been suggested as an effective cardiac preservation solution to regenerate cardiac function [61,62]; however, HTK could not fully protect against reperfusion injury during hypothermic organ preservation [63]. Since ROS generation is the main initiator of reperfusion injury, the addition of various ROS scavengers has been attempted in order to decrease reperfusion injury during heart transplantation [64,65,66]. However, the cardio-protective effect of decreasing ROS with ROS scavengers is insufficient. Since the generation of ROS and various ion transporters are involved in modulating pH during reperfusion, we thought that modulation of both ROS and ion channels is essential to decrease reperfusion injury. Therefore, the possibility of adding exogenous oh8dG, which leads to decreased ROS and modulation of NBC activity, to the preservation solution could be considered.

Our study has some experimental limitations. Cardiac function after reperfusion could not be evaluated because the Langendorff perfusion system was not used. We evaluated various cardiac injury markers to determine whether HTK leads to apoptosis during organ preservation. Although co-administration of exogenous oh8dG with HTK requires additional experiments on organ preservation for long-term exposure, the cardio-protective effect of exogenous oh8dG might provide improved beneficial effects to restore functional cardiac recovery during reperfusion.

## Data Availability

Data is contained within the article and Supplementary Material.

## Supplementary Materials

The following supporting information can be downloaded at: https://www.mdpi.com/article/10.3390/antiox11091641/s1. Figure S1: The dose-dependent cell viability of oh8dG-treated MRC5 cells; Figure S2: The effect of NBC inhibitor S0859 on H_2_O_2_-treated A549 cells; Figure S3: The dose-dependent NBC activity of oh8dG-treated A549 cells.

## References

[1] Taylor M.J., Baicu S.C. (2010). Current state of hypothermic machine perfusion preservation of organs: The clinical perspective. Cryobiology.

[2] Alves M.G., Soares A.F., Carvalho R.A., Oliveira P.J. (2011). Sodium hydrosulfide improves the protective potential of the cardioplegic histidine buffer solution. Eur. J. Pharmacol..

[3] Gomez L., Li B., Mewton N., Sanchez I., Piot C., Elbaz M., Ovize M. (2009). Inhibition of mitochondrial permeability transition pore opening: Translation to patients. Cardiovasc. Res..

[4] Garcia-Dorado D., Ruiz-Meana M., Inserte J., Rodriguez-Sinovas A., Piper H.M. (2012). Calcium-mediated cell death during myocardial reperfusion. Cardiovasc. Res..

[5] Sanada S., Komuro I., Kitakaze M. (2011). Pathophysiology of myocardial reperfusion injury: Preconditioning, postconditioning, and translational aspects of protective measures. Am. J. Physiol. Heart Circ. Physiol..

[6] Tan M.W., Sun X.D., Guo L., Su C.H., Sun X.J., Xu Z.Y. (2013). Hydrogen as additive of HTK solution fortifies myocardial preservation in grafts with prolonged cold ischemia. Int. J. Cardiol..

[7] Fridell J.A., Mangus R.S., Tector A.J. (2009). Clinical experience with histidine-tryptophan-ketoglutarate solution in abdominal organ preservation: A review of recent literature. Clin. Transplant..

[8] Latchana N., Peck J.R., Whitson B.A., Henry M.L., Elkhammas E.A., Black S.M. (2015). Preservation solutions used during abdominal transplantation: Current status and outcomes. World J. Transplant..

[9] Janssen H., Janssen P.H., Broelsch C.E. (2003). Celsior solution compared with University of Wisconsin solution (UW) and histidine-tryptophan-ketoglutarate solution (HTK) in the protection of human hepatocytes against ischemia-reperfusion injury. Transpl. Int..

[10] Semmelmann A., Neeff H., Sommer O., Thomusch O., Hopt U.T., von Dobschuetz E. (2007). Evaluation of preservation solutions by ESR-spectroscopy: Superior effects of University of Wisconsin over Histidine-Tryptophan-Ketoglutarate in reducing renal reactive oxygen species. Kidney Int..

[11] Rauen U., Klempt S., de Groot H. (2007). Histidine-induced injury to cultured liver cells, effects of histidine derivatives and of iron chelators. Cell. Mol. Life Sci..

[12] Lee E.H., Park S.R., Paik K.S., Suh C.K. (1995). Intracellular acidosis decreases the outward Na^+^-Ca^2+^ exchange current in guinea pig ventricular myocytes. Yonsei Med. J..

[13] Inserte J., Garcia-Dorado D., Ruiz-Meana M., Padilla F., Barrabes J.A., Pina P., Agullo L., Piper H.M., Soler-Soler J. (2002). Effect of inhibition of Na^+^/Ca^2+^ exchanger at the time of myocardial reperfusion on hypercontracture and cell death. Cardiovasc. Res..

[14] White C.W., Messer S.J., Large S.R., Conway J., Kim D.H., Kutsogiannis D.J., Nagendran J., Freed D.H. (2018). Transplantation of Hearts Donated after Circulatory Death. Front. Cardiovasc. Med..

[15] Leem C.H., Lagadic-Gossmann D., Vaughan-Jones R.D. (1999). Characterization of intracellular pH regulation in the guinea-pig ventricular myocyte. J. Physiol..

[16] Khandoudi N., Albadine J., Robert P., Krief S., Berrebi-Bertrand I., Martin X., Bevensee M.O., Boron W.F., Bril A. (2001). Inhibition of the cardiac electrogenic sodium bicarbonate cotransporter reduces ischemic injury. Cardiovasc. Res..

[17] Kohout T.A., Rogers T.B. (1995). Angiotensin-Ii Activates the Na^+^/HCO_3_^−^ Symport through a Phosphoinositide-Independent Mechanism in Cardiac-Cells. J. Biol. Chem..

[18] De Giusti V.C., Garciarena C.D., Aiello E.A. (2009). Role of reactive oxygen species (ROS) in angiotensin II-induced stimulation of the cardiac Na^+^/HCO_3_^−^ cotransport. J. Mol. Cell. Cardiol..

[19] De Giusti V.C., Orlowski A., Aiello E.A. (2010). Angiotensin II inhibits the electrogenic Na^+^/HCO_3_^−^ cotransport of cat cardiac myocytes. J. Mol. Cell. Cardiol..

[20] Ames B.N. (1989). Endogenous oxidative DNA damage, aging, and cancer. Free Radic. Res. Commun..

[21] Choi S., Choi H.H., Lee S.H., Ko S.H., You H.J., Ye S.K., Chung M.H. (2007). Anti-inflammatory effects of 8-hydroxy-2′-deoxyguanosine on lipopolysaccharide-induced inflammation via Rac suppression in Balb/c mice. Free Radic. Biol. Med..

[22] Kim D.H., Cho I.H., Kim H.S., Jung J.E., Kim J.E., Lee K.H., Park T., Yang Y.M., Seong S.Y., Ye S.K. (2006). Anti-inflammatory effects of 8-hydroxydeoxyguanosine in LPS-induced microglia activation: Suppression of STAT3-mediated intercellular adhesion molecule-1 expression. Exp. Mol. Med..

[23] Lee S.H., Taek Han S., Choi S.W., Sung S.Y., You H.J., Ye S.K., Chung M.H. (2009). Inhibition of Rac and Rac-linked functions by 8-oxo-2′-deoxyguanosine in murine macrophages. Free Radic. Res..

[24] Lee J.K., Ko S.H., Ye S.K., Chung M.H. (2013). 8-Oxo-2′-deoxyguanosine ameliorates UVB-induced skin damage in hairless mice by scavenging reactive oxygen species and inhibiting MMP expression. J. Dermatol. Sci..

[25] Kroese L.J., Scheffer P.G. (2014). 8-hydroxy-2′-deoxyguanosine and cardiovascular disease: A systematic review. Curr. Atheroscler. Rep..

[26] Shin S.K., Kim K.O., Kim S.H., Kwon O.S., Choi C.S., Jeong S.H., Kim Y.S., Kim J.H., Chung M.H. (2020). Exogenous 8-hydroxydeoxyguanosine ameliorates liver fibrosis through the inhibition of Rac1-NADPH oxidase signaling. J. Gastroenterol. Hepatol..

[27] Urbaniak S.K., Boguszewska K., Szewczuk M., Kazmierczak-Baranska J., Karwowski B.T. (2020). 8-Oxo-7,8-Dihydro-2′-Deoxyguanosine (8-oxodG) and 8-Hydroxy-2′-Deoxyguanosine (8-OHdG) as a Potential Biomarker for Gestational Diabetes Mellitus (GDM) Development. Molecules.

[28] Kim D.H., Im S.T., Yoon J.Y., Kim S., Kim M.K., Chung M.H., Park C.K. (2021). Comparison of therapeutic effects between topical 8-oxo-2′-deoxyguanosine and corticosteroid in ocular alkali burn model. Sci. Rep..

[29] Kim H.S., Ye S.K., Cho I.H., Jung J.E., Kim D.H., Choi S., Kim Y.S., Park C.G., Kim T.Y., Lee J.W. (2006). 8-hydroxydeoxyguanosine suppresses NO production and COX-2 activity via Rac1/STATs signaling in LPS-induced brain microglia. Free Radical. Bio. Med..

[30] Ro J.Y., Kim D.Y., Lee S.H., Park J.W., Chung M.H. (2009). Effects of 7,8-dihydro-8-oxo-deoxyguanosine on antigen challenge in ovalbumin-sensitized mice may be mediated by suppression of Rac. Br. J. Pharmacol..

[31] Hajas G., Bacsi A., Aguilera-Aguirre L., Hegde M.L., Tapas K.H., Sur S., Radak Z., Ba X., Boldogh I. (2013). 8-Oxoguanine DNA glycosylase-1 links DNA repair to cellular signaling via the activation of the small GTPase Rac1. Free Radic. Biol. Med..

[32] Chernikov A.V., Gudkov S.V., Usacheva A.M., Bruskov V.I. (2017). Exogenous 8-Oxo-7,8-dihydro-2′-deoxyguanosine: Biomedical Properties, Mechanisms of Action, and Therapeutic Potential. Biochemistry.

[33] Im S.T., Kim H.Y., Yoon J.Y., Oh J.Y., Kim M.K., Chung M.H., Paik H.J., Kim D.H. (2018). Therapeutic Effects of Topical 8-Oxo-2′-deoxyguanosine on Ethanol-Induced Ocular Chemical Injury Models. Cornea.

[34] Ock C.Y., Hong K.S., Choi K.S., Chung M.H., Kim Y., Kim J.H., Hahm K.B. (2011). A novel approach for stress-induced gastritis based on paradoxical anti-oxidative and anti-inflammatory action of exogenous 8-hydroxydeoxyguanosine. Biochem. Pharmacol..

[35] Ko S.H., Lee J.K., Lee H.J., Ye S.K., Kim H.S., Chung M.H. (2014). 8-Oxo-2′-deoxyguanosine ameliorates features of metabolic syndrome in obese mice. Biochem. Biophys. Res. Commun..

[36] Kim J.E., Hyun J.W., Hayakawa H., Choi S., Choi J., Chung M.H. (2006). Exogenous 8-oxo-dG is not utilized for nucleotide synthesis but enhances the accumulation of 8-oxo-Gua in DNA through error-prone DNA synthesis. Mutat. Res..

[37] Kim J.E., Chung M.H. (2006). 8-Oxo-7,8-dihydro-2′-deoxyguanosine is not salvaged for DNA synthesis in human leukemic U937 cells. Free Radic. Res..

[38] Ji M., In Lee S., Lee S.A., Son K.H., Hong J.H. (2019). Enhanced Activity by NKCC1 and Slc26a6 Mediates Acidic pH and Cl^−^ Movement after Cardioplegia-Induced Arrest of db/db Diabetic Heart. Mediat. Inflamm..

[39] Ackers-Johnson M., Li P.Y., Holmes A.P., O’Brien S.M., Pavlovic D., Foo R.S. (2016). A Simplified, Langendorff-Free Method for Concomitant Isolation of Viable Cardiac Myocytes and Nonmyocytes From the Adult Mouse Heart. Circ. Res..

[40] Lee Y.S., Lee H.S., Park M.K., Hwang E.S., Park E.M., Kasai H., Chung M.H. (1993). Identification of 8-Hydroxyguanine Glycosylase Activity in Mammalian-Tissues Using 8-Hydroxyguanine Specific Monoclonal-Antibody. Biochem. Biophys. Res. Commun..

[41] Borradaile N.M., Han X., Harp J.D., Gale S.E., Ory D.S., Schaffer J.E. (2006). Disruption of endoplasmic reticulum structure and integrity in lipotoxic cell death. J. Lipid Res..

[42] Yeh C.H., Chen T.P., Wang Y.C., Lin Y.M., Fang S.W. (2010). AMP-activated protein kinase activation during cardioplegia-induced hypoxia/reoxygenation injury attenuates cardiomyocytic apoptosis via reduction of endoplasmic reticulum stress. Mediat. Inflamm..

[43] Feng J., Bianchi C., Li J., Sellke F.W. (2004). Improved profile of bad phosphorylation and caspase 3 activation after blood versus crystalloid cardioplegia. Ann. Thorac. Surg..

[44] Park S.R., Lee J.W., Kim S.K., Yu W.J., Lee S.J., Kim D., Kim K.W., Jung J.W., Hong I.S. (2021). The impact of fine particulate matter (PM) on various beneficial functions of human endometrial stem cells through its key regulator SERPINB2. Exp. Mol. Med..

[45] Chen M.M., Lam A., Abraham J.A., Schreiner G.F., Joly A.H. (2000). CTGF expression is induced by TGF-beta in cardiac fibroblasts and cardiac myocytes: A potential role in heart fibrosis. J. Mol. Cell. Cardiol..

[46] Accornero F., van Berlo J.H., Correll R.N., Elrod J.W., Sargent M.A., York A., Rabinowitz J.E., Leask A., Molkentin J.D. (2015). Genetic Analysis of Connective Tissue Growth Factor as an Effector of Transforming Growth Factor beta Signaling and Cardiac Remodeling. Mol. Cell. Biol..

[47] Lin L., Kim S.C., Wang Y., Gupta S., Davis B., Simon S.I., Torre-Amione G., Knowlton A.A. (2007). HSP60 in heart failure: Abnormal distribution and role in cardiac myocyte apoptosis. Am. J. Physiol. Heart Circ. Physiol..

[48] Aredia F., Scovassi A.I. (2014). Poly(ADP-ribose): A signaling molecule in different paradigms of cell death. Biochem. Pharmacol..

[49] Ji M., Ryu H.J., Baek H.M., Shin D.M., Hong J.H. (2022). Dynamic synovial fibroblasts are modulated by NBCn1 as a potential target in rheumatoid arthritis. Exp. Mol. Med..

[50] McDonald P.C., Swayampakula M., Dedhar S. (2018). Coordinated Regulation of Metabolic Transporters and Migration/Invasion by Carbonic Anhydrase IX. Metabolites.

[51] De Giusti V.C., Ciancio M.C., Orlowski A., Aiello E.A. (2013). Modulation of the cardiac sodium/bicarbonate cotransporter by the renin angiotensin aldosterone system: Pathophysiological consequences. Front. Physiol..

[52] Zima A.V., Blatter L.A. (2006). Redox regulation of cardiac calcium channels and transporters. Cardiovasc. Res..

[53] Hyun J.W., Yoon S.H., Yu Y., Han C.S., Park J.S., Kim H.S., Lee S.J., Lee Y.S., You H.J., Chung M.H. (2006). Oh8dG induces G1 arrest in a human acute leukemia cell line by upregulating P21 and blocking the RAS to ERK signaling pathway. Int. J. Cancer.

[54] Eller-Borges R., Batista W.L., da Costa P.E., Tokikawa R., Curcio M.F., Strumillo S.T., Sartori A., Moraes M.S., de Oliveira G.A., Taha M.O. (2015). Ras, Rac1, and phosphatidylinositol-3-kinase (PI3K) signaling in nitric oxide induced endothelial cell migration. Nitric Oxide.

[55] Yu M.H., Lin M.C., Huang C.N., Chan K.C., Wang C.J. (2018). Acarbose inhibits the proliferation and migration of vascular smooth muscle cells via targeting Ras signaling. Vasc. Pharmacol..

[56] Zhan H., Bhattacharya S., Cai H., Iglesias P.A., Huang C.H., Devreotes P.N. (2020). An Excitable Ras/PI3K/ERK Signaling Network Controls Migration and Oncogenic Transformation in Epithelial Cells. Dev. Cell.

[57] Pan Y.H., Chen J., Sun C., Ma J.F., Li X. (2021). Effect of Ras-guanine nucleotide release factor 1-mediated H-Ras/ERK signaling pathway on glioma. Brain Res..

[58] Boedtkjer E., Bentzon J.F., Dam V.S., Aalkjaer C. (2016). Na^+^, HCO_3_^−^—Cotransporter NBCn1 increases pHi gradients, filopodia, and migration of smooth muscle cells and promotes arterial remodelling. Cardiovasc. Res..

[59] Ng F.L., Boedtkjer E., Witkowska K., Ren M., Zhang R., Tucker A., Aalkjaer C., Caulfield M.J., Ye S. (2017). Increased NBCn1 expression, Na^+^/HCO_3_^−^ co-transport and intracellular pH in human vascular smooth muscle cells with a risk allele for hypertension. Hum. Mol. Genet..

[60] Takago S., Matsumoto I., Kato H., Saito N., Ueda H., Iino K., Kimura K., Takemura H. (2020). Hypothermic Preservation of Rat Hearts Using Antifreeze Glycoprotein. Physiol. Res..

[61] Gu K., Kin S., Saitoh Y., Nosaka S., Sasaki T., Yamauchi M., Nakayama K. (1996). Cardioprotective effect of nicorandil in histidine-tryptophan-ketoglurate solution during the cold storage of isolated hearts. Transplantation.

[62] Saitoh Y., Hashimoto M., Ku K., Kin S., Nosaka S., Masumura S., Nakayama K. (2000). Heart preservation in HTK solution: Role of coronary vasculature in recovery of cardiac function. Ann. Thorac. Surg..

[63] Mohr A., Brockmann J.G., Becker F. (2020). HTK-N: Modified Histidine-Tryptophan-Ketoglutarate Solution-A Promising New Tool in Solid Organ Preservation. Int. J. Mol. Sci..

[64] Penna C., Perrelli M.G., Pagliaro P. (2013). Mitochondrial pathways, permeability transition pore, and redox signaling in cardioprotection: Therapeutic implications. Antioxid. Redox Signal..

[65] Pagliaro P., Moro F., Tullio F., Perrelli M.G., Penna C. (2011). Cardioprotective pathways during reperfusion: Focus on redox signaling and other modalities of cell signaling. Antioxid. Redox Signal..

[66] Penna C., Mancardi D., Rastaldo R., Losano G., Pagliaro P. (2007). Intermittent activation of bradykinin B2 receptors and mitochondrial KATP channels trigger cardiac postconditioning through redox signaling. Cardiovasc. Res..

