# Supplementary material for: Addition of oh8dG to Cardioplegia Attenuated Myocardial Oxidative Injury through the Inhibition of Sodium Bicarbonate Cotransporter Activity
Source: Antioxidants (Basel). 2022 Aug 24;11(9):1641. doi: 10.3390/antiox11091641 (PMC9495749; doi:10.3390/antiox11091641)
Supplement: Supplementary file 1 [file antioxidants-11-01641-s001.zip › antioxidants-1838149-supplementary.pdf]

Addition of oh8dG to cardioplegia attenuated myocardial oxidative injury through the inhibition of sodium bicarbonate cotransporter activity

MinJeong Ji [1], KukHui Son [2,*] and JeongHee Hong [1*]

Supplementary Figure S1. Ji *et al*.

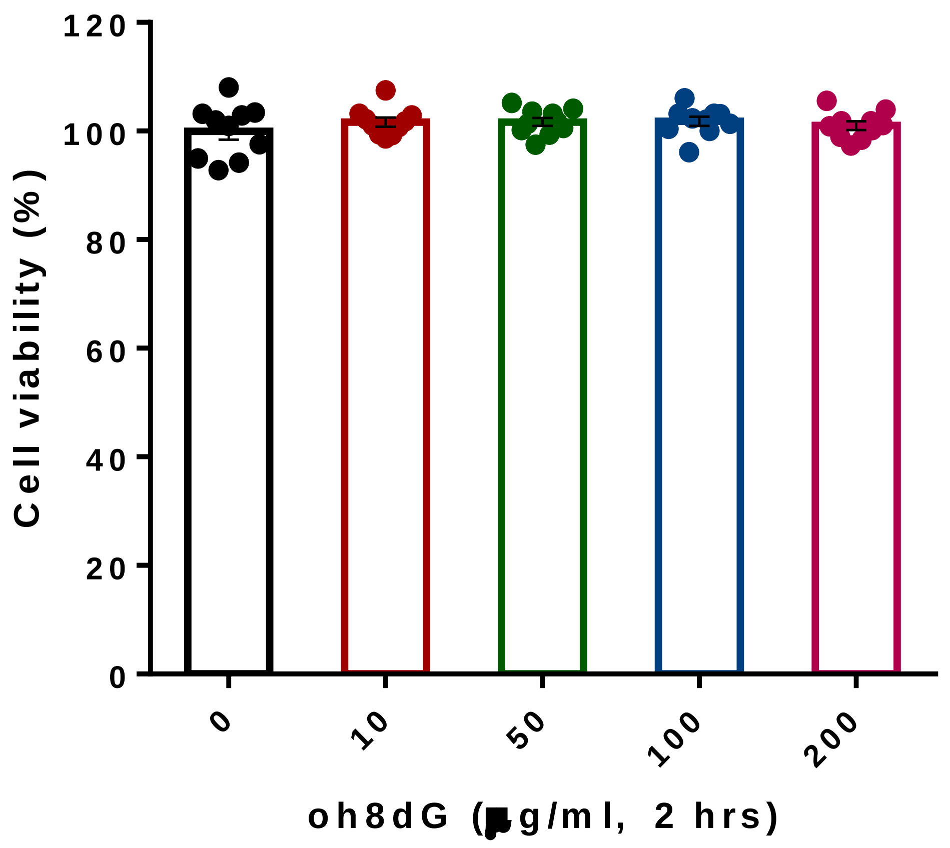

oh8dG	0 (µg/mL)	10	50	100	200
**Mean**	100	101.660	101.679	101.796	101.002
**SD**	4.907	2.578	2.362	2.616	2.487

Supplementary Figure S1. The dose-dependent cell viability of oh8dG-treated MRC5 cells

Cellular viability was measured by MTT assay.

Supplementary Figure S2. Ji *et al*.

a

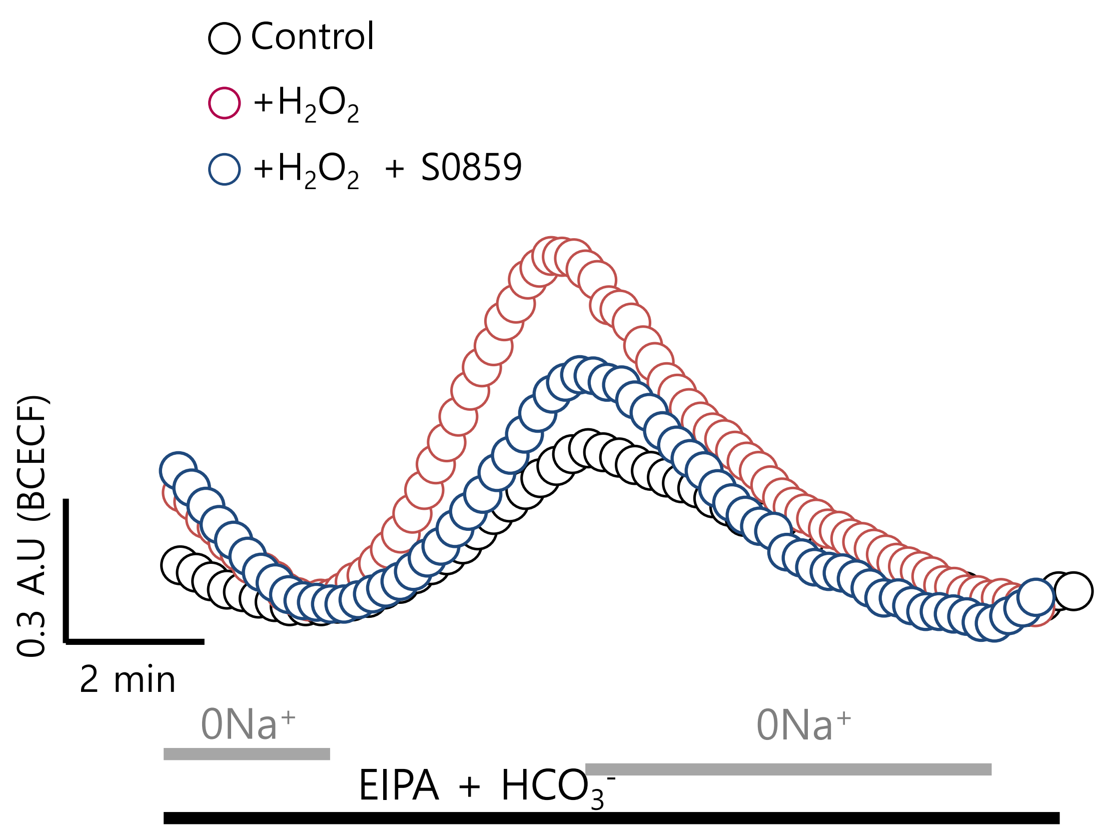

b

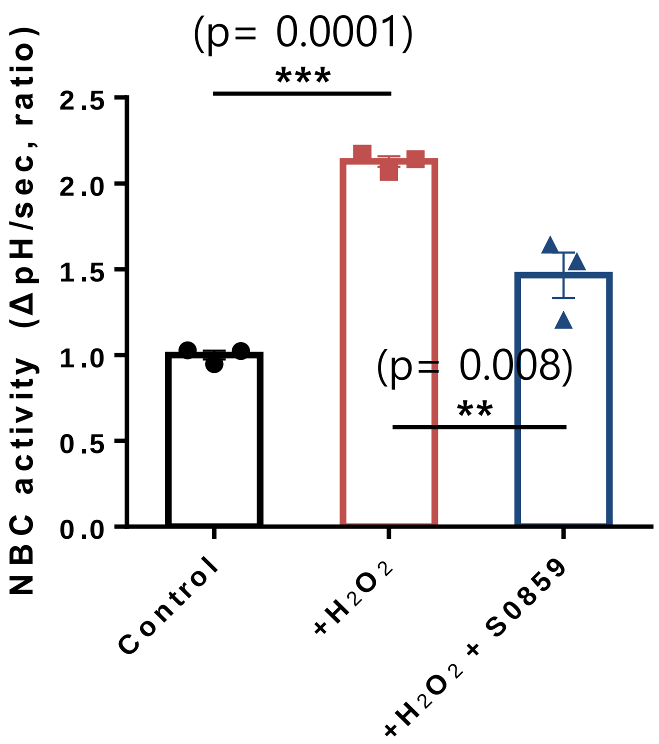

b	Mean	SD
**Control**	1	0.045
**+H_2O_2**	2.127	0.054
**+H_2O_2 + S0859**	1.464	0.229

Supplementary Figure S2. The effect of NBC inhibitor S0859 on H_2O_2-treated A549 cells

(a) NBC activity was measured by measuring changes in pH_i which incubated with H_2O_2 in the presence or absence of NBC inhibitor S0859 (40 μM) for 1 hr. **(b)** The bars present mean ± SEM (n = 3, $^{**}p < 0.01$, $^{***}p < 0.001$).

Supplementary Figure S3. Ji *et al*.

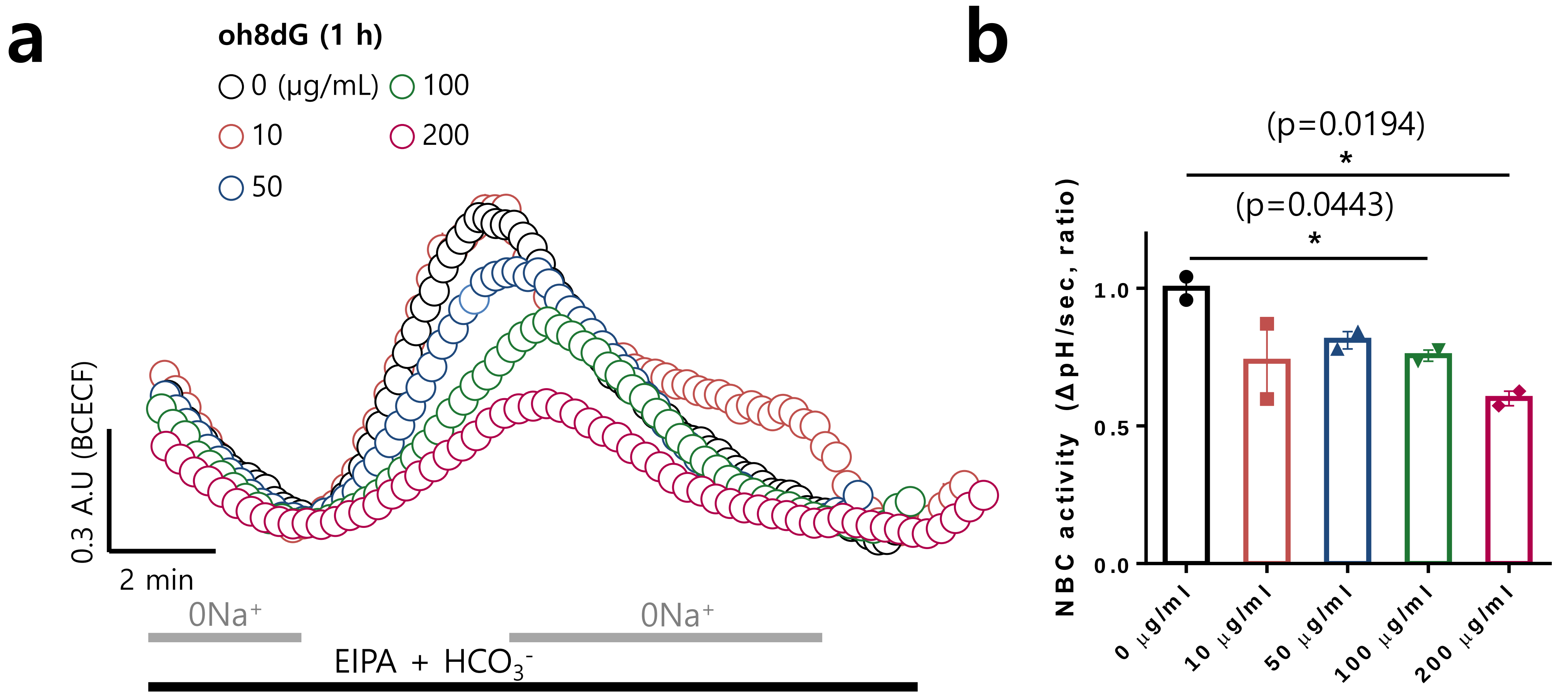

oh8dG	0 (μg/mL)	10	50	100	200
**Mean**	1	0.734	0.811	0.755	0.600
**SD**	0.059	0.193	0.044	0.028	0.037

Supplementary Figure S3. The dose-dependent NBC activity of oh8dG-treated A549 cells

(a) NBC activity was measured by measuring changes in pH_i which incubated with dose-dependent oh8dG. **(b)** The bars present mean ± SEM (n = 2, *p < 0.05).
